# Pathological Evaluation of Resected Colorectal Liver Metastases: mFOLFOX6 Plus Bevacizumab versus mFOLFOX6 Plus Cetuximab in the Phase II ATOM Trial

**DOI:** 10.3390/cancers14184392

**Published:** 2022-09-09

**Authors:** Takao Takahashi, Kazuyuki Ishida, Yasunori Emi, Michiie Sakamoto, Johji Imura, Shinichi Aishima, Kei Muro, Hiroyuki Uetake, Eiji Oki, Yu Katayose, Kazuhiro Yoshida, Michiaki Unno, Ichinosuke Hyodo, Naohiro Tomita, Kenichi Sugihara, Yoshihiko Maehara

**Affiliations:** 1 Department of Digestive Surgery, Gifu University Hospital, 1-1 Yanagido, Gifu 501-1194, Japan; 2 Department of Diagnostic Pathology, Dokkyo Medical University, 880 Kitakobayashi, Mibu 321-0293, Japan; 3 Department of Surgery, Saiseikai Fukuoka General Hospital, 1-3-46 Tenjin, Chuou-ku, Fukuoka 810-0001, Japan; 4 Department of Pathology, Keio University School of Medicine, 35 Shinanomachi, Shinjuku-ku, Tokyo 160-8582, Japan; 5 Department of Pathology, Kumagaya General Hospital, 4-5-1 Nakanishi, Kumagaya 360-8567, Japan; 6 Department of Pathology and Microbiology, Faculty of Medicine, Saga University, 5-1-1 Nabeshima, Saga 849-8501, Japan; 7 Department of Clinical Oncology, Aichi Cancer Center Hospital, 1-1 Kanoko-den Chikusa-ku, Nagoya 464-8681, Japan; 8 Department of Clinical Research, National Disaster Medical Center, 3256 Midoricho, Tachikawa 190-0014, Japan; 9 Department of Surgery and Science, Graduate School of Medical Sciences, Kyushu University, 3-1-1 Maidashi, Higashi-ku, Fukuoka 812-8582, Japan; 10 Hepato-Biliary and Pancreatic Surgery, Tohoku Medical and Pharmaceutical University, 4-4-1 Komatsushima, Aobaku, Sendai 981-8558, Japan; 11 Department of Surgery, Tohoku University Graduate School of Medicine, 2-1 Seiryo-machi, Aoba-ku, Sendai 980-8575, Japan; 12 Department of Gastrointestinal Medical Oncology, National Hospital Organization Shikoku Cancer Center, 160 Kou, Minamiumemoto-machi, Matsuyama 791-0280, Japan; 13 Cancer Treatment Center, Toyonaka Municipal Hospital, 4 Chome-14-1 Shibaharacho, Toyonaka 560-8565, Japan; 14 Department of Surgical Oncology and Gastroenterology, Tokyo Medical and Dental University, 1-5-45 Yushima, Bunkyo-ku, Tokyo 113-8510, Japan; 15 Department of Surgery, Kyushu Central Hospital of the Mutual Aid Association of Public-School Teachers, 3-23-1 Shiobara, Minami-ku, Fukuoka 815-8588, Japan

**Keywords:** ATOM trial, bevacizumab, cetuximab, chemotherapy, colorectal liver metastases, histopathological response

## Abstract

**Simple Summary:**

We compared the pre-planned histopathological responses, such as tumor regression grade (TRG), modified TRG, and dangerous halo (DH) of resected liver metastases, in patients who received modified FOLFOX6 plus bevacizumab and modified FOLFOX6 plus cetuximab for liver-limited colorectal metastases from the ATOM trial. We clarified the difference between bevacizumab and cetuximab in terms of histological response. TRG is a useful marker for determining prognosis in both treatments. We also showed, for the first time, that DH is associated with prognosis.

**Abstract:**

We compared the preplanned histopathological responses of resected liver metastases from patients who received modified FOLFOX6 plus bevacizumab or modified FOLFOX6 plus cetuximab for liver-limited colorectal metastases in the ATOM trial. Fibrosis and viable tumor cells in tumor regression grade (TRG), infarct-like necrosis in modified TRG (mTRG), and dangerous halo (DH) were assessed. Fifty-five patients (28 and 27 patients in the bevacizumab and cetuximab arms, respectively) were divided into the low (viable tumor cells ≤ 50%) and high (>50%) TRG or mTRG groups. DH was characterized as absent/rare or focal/diffuse. Compared to the bevacizumab arm, the cetuximab arm was more effective, with respect to low TRG (13 vs. 23 patients) and absent/rare DH (14 vs. 19 patients), respectively. Low mTRG was similarly observed in both arms. Low TRG/mTRG and absent/rare DH showed better relapse-free survival (RFS) than high TRG/mTRG and focal/diffuse DH. In the bevacizumab arm, a significant difference in RFS existed between the low and high TRG groups, while in the cetuximab arm, for TRG, mTRG, and DH, the low and absent/rare groups demonstrated significantly longer RFS than the high and focal/diffuse groups, respectively. TRG could estimate RFS in patients who underwent liver metastasectomy after bevacizumab or cetuximab chemotherapy.

## 1. Introduction

Colorectal cancer (CRC) is the third most common cancer and second leading cause of cancer-related deaths worldwide [1]. Colorectal liver metastases (CRLM) are present in 56% of patients with stage IV CRC, and liver recurrence occurs in almost 7% of patients after curative resection of CRC [2]. Therefore, strategies that improve the outcomes for patients with CRLM are needed. Subject to the efficacy of neoadjuvant chemotherapy, resection is occasionally performed for patients with initially unresectable/borderline resectable CRLM, which can contribute to long-term survival [3,4]. Promising outcomes have been reported for molecular targeted treatment of CRLM that target either the epidermal growth factor receptor (EGFR) or vascular endothelial growth factor (VEGF) [5,6,7,8]. Results from our group’s ATOM phase II trial revealed similar efficacy between mFOLFOX6 plus bevacizumab (BEV) and mFOLFOX6 plus cetuximab (CET) in patients with wild-type *KRAS/RAS* liver-limited CRLM; however, CET provided a superior response rate [9].

Chemotherapy efficacy is generally evaluated from radiological response (i.e., CRLM size reduction), according to the Response Evaluation Criteria in Solid Tumors (RECIST); however, several reports have indicated that histopathological response or characteristic histological findings in resected specimens of liver metastases are important, evaluated according to tumor regression grade (TRG) [10], modified tumor regression grade (mTRG) [11], dangerous halo (DH) [12], and sinusoidal obstruction syndrome (SOS) systems [13,14]. As an antibody to VEGF, BEV reportedly exerts an anti-angiogenic effect and specific morphological tumor response, enhancing tumor necrosis and increasing survival [15,16]. Klinger et al. reported that the addition of BEV improved the pathological response compared to XELOX/FOLFOX alone, leading to progression-free survival (PFS) and overall survival (OS) prolongation [17]. In addition, BEV can reduce the incidence and severity of SOS histology treated with oxaliplatin-based chemotherapy in non-tumorous liver tissues [14,18]. However, there are few reports on the pathological response to CET. In addition, there have been no reports of randomized controlled trials regarding histopathological changes induced by BEV or CET for non-optimally resectable CRLM. Therefore, we performed a histopathological evaluation of CRLM specimens from the ATOM trial [9], comparing preoperative treatment using mFOLFOX6 plus BEV and mFOLFOX6 plus CET.

## 2. Methods

### 2.1. Patients

Patients and methods from the ATOM trial have been described elsewhere [9]. Written informed consent was provided by participants. The trial protocol was approved by the ethics committee at each participating center and the study was performed at 63 Japanese institutions (ClinicalTrials.gov, NCT01836653; University Hospital Medical Information Network Clinical Trials Registry, UMIN000010209).

### 2.2. Endpoints

The primary endpoint was the PFS. Relapse was considered a PFS event for patients who underwent liver resection after the protocol treatment. The secondary endpoints were the response rate, tumor shrinkage at week 8, liver resection rate, OS, quality of life, and adverse events; we have previously reported these results [9].

Preplanned pathological assessments were performed using resected liver specimens and evaluations based on TRG [10], mTRG [11], DH [12], and SOS systems [13,14]; these analyses aimed to clarify differences in treatment arms and investigate whether these pathological markers could predict relapse-free survival (RFS) and OS.

### 2.3. Pathological Assessment and Radiological Assessment

Following the pathological assessment procedure in the protocol, we reviewed all available specimens of the CRLM and non-CRLM areas, which were formalin-fixed, paraffin-embedded, and stained with hematoxylin and eosin at each institution. Up to three radiologically defined target CRLMs, including the largest nodule, were collected for pathological assessment. TRG, mTRG, and DH were pathologically evaluated in all specimens of the largest section of CRLM. SOS was evaluated using specimens in which the CRLM was not present in the same section. If no such specimen was available, SOS was evaluated in an area distant from the CRLM, which was pathologically judged to be unaffected by the CRLM. Resected liver specimens were evaluated by an independent pathological review committee (K.I., S.A., M.S., and J.I.). The raters were blinded to treatment arm and clinical outcomes.

**Figure 1:**
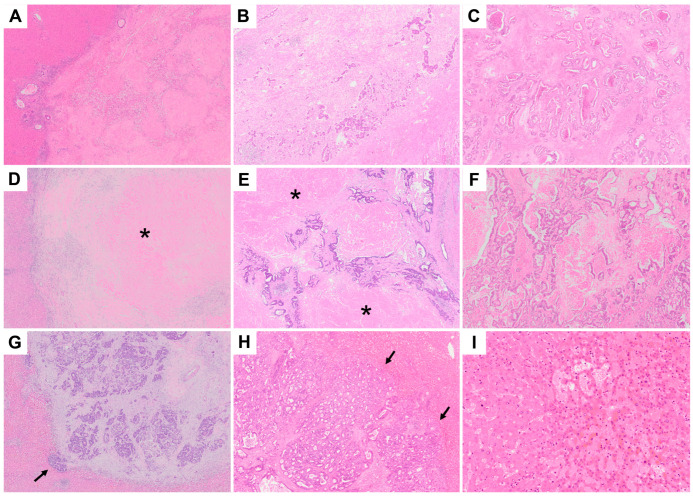
Representative histologic findings of colorectal liver metastases ((**A**–**H**), original magnification ×20; (**I**), ×100). (**A**–**C**): Tumor regression grade (TRG). (**A**–**C**) correspond to TRG2 (low grade; mostly abundant fibrosis with a small number of viable tumor cells), TRG3 (low grade; predominantly fibrotic, but with more viable tumor cells), and TRG4 (high grade; more tumor cells than fibrosis), respectively. (**D**–**F**): Modified TRG (mTRG) considering the presence of infarct-like necrosis (ILN) (asterisk). (**D**–**F**) correspond to mTRG2 (low grade; mostly abundant fibrosis and ILN with a small number of viable tumor cells), mTRG3 (low grade; mainly fibrosis and ILN, but a larger number of viable tumor cells), and mTRG4 (high grade; more tumor cells than fibrosis and ILN), respectively. (**G**): “Rare” dangerous halo (scattered tumor cells that infiltrate the liver parenchyma for <10% of the lesion’s circumference) (arrows). (**H**): “Diffuse” dangerous halo (scattered cells that infiltrate the liver parenchyma for >50% of the lesion’s circumference) (arrows). (**I**): Sinusoidal obstruction syndrome grade 2 (moderate and extended from zone 1 to zone 2), which was observed in the background only in the cetuximab arm.

Details regarding the definition of the pathological evaluation are presented in Supplementary Table S1. The presence of viable tumor cells was assessed for each patient who underwent CRLM hepatectomy, based on the TRG [10] and mTRG [11] systems. TRG uses five classifications [10], which are described in Figure 1A–C. mTRG considers the presence of infarct-like necrosis (ILN) [11]; necrosis is generally classified as usual necrosis—necrosis with nuclear debris surrounded by viable tumor cells—or ILN, caused by chemotherapy and appears as eosinophilic homogenous necrosis with no nuclear debris, surrounded by hyalinized fibrosis with histiocytic infiltration [11]. The mTRG classifications are found in Figure 1D–F. The DH is a cluster of tumor cells that infiltrate the surrounding liver parenchyma without proliferating fibrotic stroma [12]. DH classifications are described in Figure 1G,H [12]. The SOS system classifies lesions as grades 1–3. These are described in Figure 1I [13,14].

We used the Response Evaluation Criteria in Solid Tumors (RECIST) version 1.1 as the radiological response and performed tumor assessment at the baseline and tumor evaluations using computed tomography (CT) scans every 8 weeks.

### 2.4. Classification of Histopathological Response

Patients were categorized into the following two groups based on the original TRG/mTRG grades: low TRG/mTRG based on TRG/mTRG grades 1–3 (≤50% viable tumor cells) (Figure 1A,B,D,E) and high TRG/mTRG based on TRG/mTRG grades 4–5 (>50% viable tumor cells) (Figure 1C,F). DH was characterized as absent/rare (Figure 1G) and focal/diffuse (Figure 1H). Histopathological classifications according to the TRG, mTRG, DH, and SOS systems were compared between the BEV and CET arms. Histopathological responses were evaluated for correlations with RFS (time to relapse after resection) and OS to determine their prognostic marker capability; moreover, we evaluated whether histopathological responses (low TRG/mTRG or high TRG/mTRG based on the TRG/mTRG systems) were associated with radiological response to the best overall response (complete response [CR], partial response [PR], stable disease [SD], and progressive disease) based on RECIST version 1.1.

### 2.5. Statistical Analyses

Patient characteristics were compared between the BEV and CET arms using either the chi-squared or Wilcoxon’s test. Hazard ratios for RFS in both arms were estimated using a Cox proportional hazards model; survival curves were estimated using the Kaplan–Meier method and compared using the log-rank test. All statistical analyses were performed using SAS (version 9.4; SAS Institute, Cary, NC, USA).

## 3. Results

### 3.1. Patients

**Figure 2:**
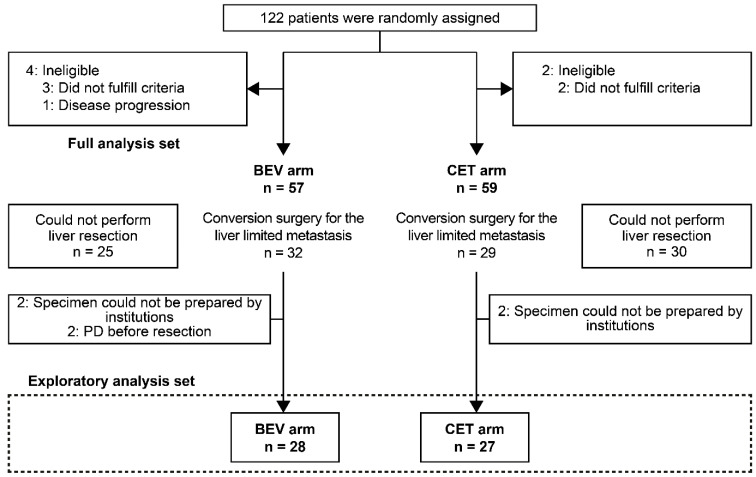
CONSORT flow diagram. BEV, bevacizumab; CET, cetuximab; PD, progressive disease.

Of the 116 patients with liver limited metastasis from colorectal cancer that were initially unresectable or difficult to be resected from the ATOM study (57 in the BEV arm and 59 in the CET arm), the conversion surgery could be performed in 32 patients in the BEV arm and 29 in the CET arm. A subgroup of 55 patients from the ATOM trial were eligible for this study (BEV arm, 28; CET arm, 27). A CONSORT flow diagram is presented in Figure 2, and patient characteristics are presented in Table 1. There were no significant differences between the BEV and CET arms. In addition, the procedures for the liver resection and the postoperative complications are described in Supplementary Tables S2 and S3, respectively. There were no significant differences in surgical procedures or postoperative complications between the BEV and CET groups.

### 3.2. Differences in TRG, mTRG, DH, and SOS

For all patients, the TRG 1/2/3/4/5 counts were 1/15/20/15/4 (1.8%/27.3%/36.4%/27.3%/7.3%), respectively. The TRG 1/2/3/4/5 counts were 0/6/7/12/3 (0%/21.4%/25.0%/42.9%/10.7%), respectively, in the BEV arm, and 1/9/13/3/1 (3.7%/33.3%/48.1%/11.1%/3.7%), respectively, in the CET arm. Meanwhile, for all patients, the mTRG 1/2/3/4/5 counts were 1/21/25/7/1 (1.8%/38.2%/45.5%/12.7%/1.8%), respectively. The mTRG 1/2/3/4/5 counts were 0/12/11/5/0 (0%/42.9%/39.3%/17.9%/0%), respectively, in the BEV arm, and 1/9/14/2/1 (3.7%/33.3%/51.9%/7.4%/3.7%), respectively, in the CET arm (Supplementary Table S4). The results after the division into low TRG/mTRG and high TRG/mTRG are shown in Table 2. BEV improved histological response with mTRG rather than TRG, because the cases that involved ILN were mainly observed in the BEV arm. TRG assessment suggested that CET provided a significantly better histopathological response than BEV (*p* = 0.003), while the mTRG assessment revealed similar proportions between the two (*p* = 0.478) (Table 2).

Among all patients, the DH absent/rare/focal/diffuse counts constituted 14/19/13/9 cases (25.5%/34.5%/23.6%/16.4%), respectively. The absent/rare/focal/diffuse counts constituted 4/10/6/8 cases (14.3%/35.7%/21.4%/28.6%), respectively, in the BEV arm, and 10/9/7/1 cases (37.0%/33.3%/25.9%/3.7%), respectively, in the CET arm (Supplementary Table S4). CET tended to be better than BEV, with a decrease in the DH classifications. However, there were no significant differences between absent/rare and focal/diffuse DH (*p* = 0.123) (Table 2).

Among all patients, SOS grade 1/2/3 counts constituted 50/5/0 cases (90.9%/9.1%/0%), respectively. Grade 1/2/3 counts constituted 28/0/0 cases (100%/0%/0%), respectively, in the BEV arm, and 22/5/0 cases (81.5%/18.5%/0%), respectively, in the CET arm (Supplementary Table S4). CET caused SOS, but BEV was thought to have prevented SOS significantly (*p* = 0.017) (Table 2). However, this did not affect postoperative complications (Supplementary Table S3).

### 3.3. Relationship between Histological Response Based on TRG/mTRG and Radiological Response According to RECIST (Version 1.1)

These data are contained in Supplementary Tables S5 and S6. In the CET arm, all patients who underwent liver resection achieved a radiological response of PR, despite high TRG and mTRG findings in four (14.8%) and three cases (11.1%), respectively. However, in the BEV arm, eight patients who achieved a radiological response of SD did not exhibit low TRG findings, whereas seven had low mTRG findings (Supplementary Tables S5 and S6). In the BEV arm, there were cases in which low mTRG was observed by mTRG assessment, although the patients achieved a radiological response of SD.

### 3.4. TRG and mTRG Classifications as Predictors of RFS and OS

**Figure 3:**
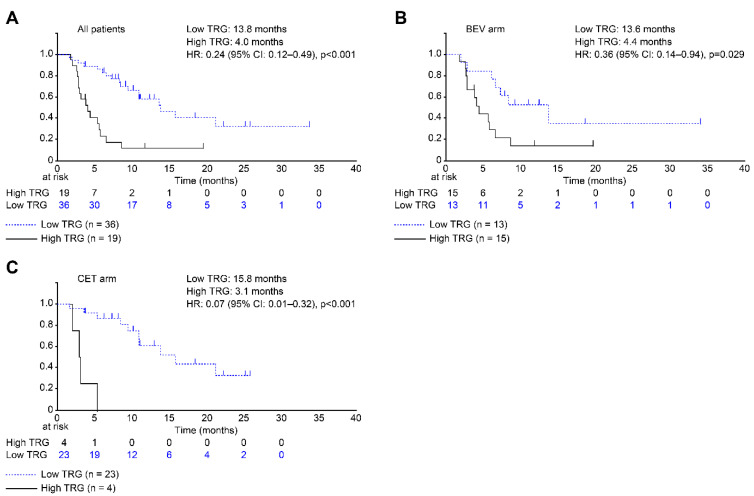
Kaplan–Meier curves for recurrence-free survival (RFS, time to relapse after resection) based on the tumor regression grade (TRG). Curves are shown for all patients (**A**), patients in the bevacizumab (BEV) arm (**B**), and patients in the cetuximab (CET) arm (**C**). Relative to patients with high TRG, those with low TRG had significantly better RFS (median: 4.0 vs. 13.8 months, hazard ratio (HR): 0.24; 95 confidence interval (CI): 0.12–0.49; *p* < 0.001). Similarly, relative to high TRG, low TRG was associated with significantly better RFS in the BEV arm (median: 4.4 vs. 13.6 months; HR: 0.36; 95% CI: 0.14–0.94; *p* = 0.029) and in the CET arm (median: 3.1 vs. 15.8 months; HR: 0.07; 95% CI: 0.01–0.32; *p* < 0.001).

Figure 3 illustrates the Kaplan–Meier curves for RFS based on the TRG system. In addition, the Kaplan–Meier curve of RFS based on mTRG is shown in Supplementary Figure S1. Patients with low TRG had significantly longer RFS than those with high TRG based on both systems (hazard ratio (HR): 0.24 (95% confidence interval (CI): 0.12–0.49), *p* < 0.001) (Figure 3A). Patients with low mTRG also had significantly longer RFS than those with high mTRG based on both systems (HR: 0.34 (95% CI: 0.13–0.85); *p* = 0.015) (Supplementary Figure S1A). Additionally, patients in the CET arm with low TRG/mTRG had significantly longer RFS than patients with high TRG/mTRG based on both systems (HR: 0.07 (95% CI: 0.01–0.32); *p* < 0.001; and HR: 0.11 (95% CI: 0.02–0.52); *p* = 0.001, respectively) (Figure 3C and Figure S1C). However, in the BEV arm, low TRG was associated with significantly longer RFS based on the TRG system alone (HR: 0.36 (95% CI: 0.14–0.94); *p* = 0.029) (Figure 3B and Figure S1B).

Patients with low TRG/mTRG had significantly longer OS than those with high TRG/mTRG, based on both systems (*p* = 0.001 and *p* = 0.010, respectively) (Supplementary Figures S2A and S3A). Nevertheless, in the CET arm, a significant increase in OS was only associated with low TRG based on the TRG system (Supplementary Figure S2C). Although the relationships were not statistically significant, an increase in OS tended to be associated with low mTRG in the CET arm, based on the mTRG system, and in the BEV arm, based on both systems (Supplementary Figures S2B and S3B,C).

### 3.5. DH Classifications as Predictors of RFS and OS

**Figure 4:**
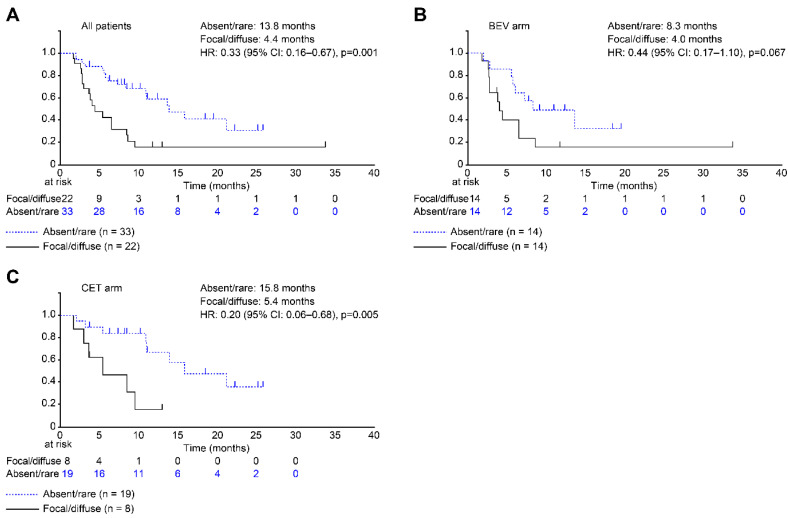
Kaplan–Meier curves for relapse-free survival (RFS, time to relapse after resection) according to the dangerous halo (DH) classification. Curves are shown for all patients (**A**), those in the bevacizumab (BEV) arm (**B**), and those in the cetuximab (CET) arm (**C**). Relative to the patients in the focal/diffuse classification, the patients in the absent/rare classification had significantly better RFS (median: 4.4 vs. 13.8 months; hazard ratio (HR): 0.33; 95% confidence interval (CI): 0.16–0.67; *p* = 0.001). Similarly, relative to the focal/diffuse classification, the absent/rare classification was associated with significantly better RFS in the BEV arm (median: 4.0 vs. 8.3 months; HR: 0.44; 95% CI: 0.17–1.10; *p* = 0.067) and in the CET arm (median: 5.4 vs. 15.8 months; HR: 0.20; 95% CI: 0.06–0.68; *p* = 0.005).

Figure 4 shows the Kaplan–Meier curves for RFS according to the DH classification. Patients with absent/rare DH had significantly longer RFS than those with the focal/diffuse classification (HR: 0.33 (95% CI: 0.16–0.67); *p* = 0.001). Only patients in the CET arm with absent/rare DH had significantly longer RFS than those with the focal/diffuse classification (HR: 0.20 (95% CI: 0.06–0.68); *p* = 0.005). However, no significant differences in the OS were observed.

## 4. Discussion

To our knowledge, this is the first report to involve a preplanned evaluation of histopathological changes induced by both anti-VEGF and anti-EGFR treatment in previously untreated CRLM cases using prospectively collected data, which were obtained from the ATOM trial. As a VEGF-targeting treatment, BEV induces an anti-angiogenic effect and a specific morphological tumor response that enhances tumor necrosis and increases the survival benefit after chemotherapy [15,16]. Furthermore, BEV significantly improves tumor regression in response to chemotherapy, with improvement in the histological response leading to significant increases in PFS and OS [17,19,20]. A previous trial on patients with CRLM revealed that CET (an anti-EGFR treatment) also provided a response rate improvement and high liver resection rates [5]. No randomized controlled trials have compared BEV and CET for treating unresectable CRLM. Therefore, it is unclear whether either drug provided a superior histopathological response in liver-limited CRLM. This study revealed differences in the tumor regression histopathological patterns of the BEV and CET arms of the ATOM trial. The CET arm had better histopathological responses than the BEV arm in the TRG system. However, using the mTRG system incorporated the increased ILN in the BEV arm, causing similar histopathological responses in both arms. In a previous study, BEV had a higher histological response with mTRG evaluation [11]. It is not known whether either chemotherapy or targeted drugs influenced DH classification in this setting, although we found that CET positively influenced DH classification in CRLM. Furthermore, the BEV arm had less severe SOS [14,18]; however, 18.5% of patients in the CET arm had grade 2 SOS. However, SOS grading was not related to postoperative complications after hepatectomy. A high rate of SOS is associated with the use of oxaliplatin-containing regimens (including FOLFOX) [13], although SOS can be prevented by incorporating BEV [14,18].

Rubbia-Brandt et al. [10] used the TRG system to histopathologically evaluate the response of CRLM to treatment, and reported that both the histopathological response and TRG classification were independent predictors of disease-free survival and OS in patients who received neoadjuvant chemotherapy. Another report indicated that ILN (mTRG) was associated with disease-free survival after BEV treatment, with mTRG classification being a prognostic factor [11]. We used both TRG and mTRG systems to evaluate the histopathological responses; moreover, we analyzed whether these responses predicted RFS and OS among all patients in the BEV and/or CET arms. We classified the responses as either TRG/mTRG 1–3 as low TRG/mTRG or TRG/mTRG 1–2 as low TRG/mTRG, and observed that TRG/mTRG 1–3 as low TRG/mTRG predicted good OS and RFS among patients who underwent CRLM resection. Additionally, low TRG based only on the TRG system predicted good RFS in both BEV and CET arms. No significant difference in RFS was observed between low mTRG and high mTRG based on the mTRG system in the BEV arm. Moreover, low TRG based on the TRG system was only associated with significantly better OS in the CET arm. Therefore, we suggest that prognosis using the histopathological response to targeted treatment should be based on the TRG system.

This study also revealed that DH classification of the resected CRLM specimens varied between the two arms. To our knowledge, this is the first study to report that DH classification predicted RFS in this setting, with the absent/rare classification being associated with significantly longer RFS. Moreover, the CET arm had better DH classifications than the BEV arm.

There were differences in the BEV and CET arms between the histopathological (evaluated using the TRG and mTRG systems) and radiological responses (evaluated using the RECIST system). In the CET arm, approximately 10% of patients had high TRG/mTRG, based on both the TRG and mTRG systems, although the radiological response indicated PR. In the BEV arm, low TRG based on the TRG system was not observed in patients with SD, although low mTRG based on the mTRG system was observed in approximately 30% of patients with SD. Previous reports have indicated that the morphological response to preoperative chemotherapy is an independent prognostic factor in patients who undergo CRLM resection [15,16,21]. Furthermore, a BEV-containing regimen provided higher optimal morphological response rates—relative to chemotherapy alone—suggesting that BEV contributes to optimal morphological responses in CRLM [15,16,21]. Morphological responses are also superior to the RECIST-based response for predicting both histopathological response and survival [21]. In the BEV arm, seven of eight patients with SD (88%) had low mTRG based on the mTRG system, which suggests that the mTRG system might be more useful for evaluating the histopathological response to BEV. However, the CET arm also had good histopathological responses based on both the TRG and mTRG systems. Therefore, our results indicate that the TRG system is superior to the mTRG system for predicting RFS after regimens, including BEV or CET. We also observed that CET treatment tended to provide a greater decrease in DH classification, relative to BEV treatment.

The median RFS interval calculations began at surgical resection and were 6.5 and 13.8 months in the BEV and CET arms, respectively (HR: 0.576 (95% CI: 0.286–1.157), *p* = 0.1155) (Supplementary Figure S4). The CET arm tended to have better RFS than the BEV arm. This could be attributed to the pathological responses of TRG and DH, which were better in the CET arm than in the BEV arm. These results suggested that the CET treatment may exhibit better PFS, which was the primary endpoint in the ATOM trial.

Cremolini et al. [22] reported that BEV could induce a better histopathological response than CET when combined with the FOLFOXIRI regimen; however, the aforementioned study had its limitations. The data were extracted from various clinical trials, including the TRIBE and MACBETH trials. Moreover, patients were not randomized to receive either chemotherapy with BEV or CET. Another report indicated that BEV retrospectively induced significantly better histopathological responses than CET [23]. In another study, the histological responses of BEV and CET were compared to their respective borderline resectable CRLMs, and the histological responses of BEV and CET were found to be similar; however, BEV caused high necrosis and CET caused high fibrosis. Each molecular-target drug was characterized by a histological response [24]. In this study, CET showed a better histological response than BEV in hepatectomy (conversion surgery) for initial unresectable/borderline resectable CRLM. BEV may also be most effective in terms of histological effects, when combined with high cell-mediated drugs, such as in triplet therapy. BEV and CET may have different types of histological responses.

This study had some limitations. Of the 116 patients in the ATOM trial who were initially unresectable or difficult to resect, 61 underwent hepatectomy. Of these, only 55 excised specimens that met the eligibility criteria were assessed in the study. Accordingly, this study was limited by its small sample size; therefore, there was insufficient statistical power to compare two biological agents. Moreover, the OS data were immature, and a longer-term follow-up period is required. However, four pathological specialists evaluated the pathological response in detail using these excised specimens.

## 5. Conclusions

The pathological responses evaluated using the TRG, mTRG, and DH systems were significantly associated with RFS in patients who underwent CRLM resection, after receiving either mFOLFOX6 plus BEV or mFOLFOX6 plus CET. TRG assessment revealed that the CET arm induced a significantly better histopathological response than the BEV arm, while the mTRG assessment revealed similar proportions between the CET and BEV arms. The CET arm had greater decreases in DH classification than the BEV arm. This result may indicate that the CET arm tended to have better RFS than the BEV arm in liver resection cases. The TRG system may be a more useful prognostic marker for evaluating the response to BEV- or CET-containing chemotherapy, before performing CRLM resection.

## Figures and Tables

**Table 1:** Patient characteristics.

Characteristic		BEV Arm (*n* = 28)	CET Arm (*n* = 27)	*p*-Value
Age (years)	Median (range)	61.0 (32.0–79.0)	63.0 (50.0–77.0)	0.316
Sex	Male	16 (57.1%)	17 (63.0%)	0.660
	Female	12 (42.9%)	10 (37.0%)	
ECOG PS	0	25 (89.3%)	25 (92.6%)	0.670
	1	3 (10.7%)	2 (7.4%)	
Adjuvant chemotherapy	Yes	3 (10.7%)	2 (7.4%)	0.670
Prior oxaliplatin	Yes	1 (3.6%)	2 (7.4%)	0.531
Tumor location	Right	3 (10.7%)	8 (29.6%)	0.080
	Left	25 (89.3%)	19 (70.4%)	
Tumor status	Synchronous, with primary tumor	4 (14.3%)	5 (18.5%)	0.476
	Synchronous, without primary tumor	19 (67.9%)	19 (70.4%)	
	Metachronous	5 (17.9%)	3 (11.1%)	
Number of liver metastases (at the time of registration)	1–4	10 (35.7%)	13 (48.1%)	0.350
	≥5	18 (64.3%)	14 (51.9%)	
Diameter of liver metastases (at the time of registration)	≤5 cm	10 (35.7%)	10 (37.0%)	0.919
	>5 cm	18 (64.3%)	17 (63.0%)	
Chemotherapy course up to hepatectomy	Median (range)	8 (6–22)	8 (4–31)	0.1088
Period from registration to hepatectomy (days)	Median (range)	160 (116–439)	158 (92–465)	0.7173
Number of liver metastases before hepatectomy	Median (range)	6 (1–15)	4 (1–18)	0.8458

BEV, bevacizumab; CET, cetuximab; ECOG PS, Eastern Cooperative Oncology Group performance status.

**Table 2:** Pathological responses of the TRG/mTRG systems and DH/SOS classifications between the BEV and CET arms.

Pathological Response		All Patients (*n* = 55)	BEV Arm (*n* = 28)	CET Arm (*n* = 27)	*p*-Value
TRG	Low TRG (TRG 1–3)	36 (65.5%)	13 (46.4%)	23 (85.2%)	0.003
	High TRG (TRG 4–5)	19 (34.5%)	15 (53.6%)	4 (14.8%)	
mTRG	Low mTRG (mTRG 1–3)	47 (85.5%)	23 (82.1%)	24 (88.9%)	0.478
	High mTRG (mTRG 4–5)	8 (14.5%)	5 (17.9%)	3 (11.1%)	
DH	Absent/rare	33 (60.0%)	14 (50.0%)	19 (70.4%)	0.123
	Focal/diffuse	22 (40.0%)	14 (50.0%)	8 (29.6%)	
SOS	Grade 1	50 (90.9%)	28 (100%)	22 (81.5%)	0.017
	Grade 2/3	5 (9.1%)	0	5 (18.5%)	

BEV, bevacizumab; CET, cetuximab; DH, dangerous halo; mTRG, modified tumor regression grade; SOS, sinusoidal obstruction syndrome; TRG, tumor regression grade.

## Data Availability

The data sets used and/or analyzed during the current study are available from the corresponding author upon reasonable request.

## Supplementary Materials

The following supporting information can be downloaded at: https://www.mdpi.com/article/10.3390/cancers14184392/s1, “*Cancers*” website, Figure S1: Kaplan–Meier curves for relapse-free survival (RFS, time to relapse after resection) according to the modified tumor regression grade (mTRG); Figure S2: Kaplan–Meier curves for overall survival (OS) based on the tumor regression grade (TRG); Figure S3: Kaplan–Meier curves for overall survival (OS) based on the modified tumor regression grade (mTRG); Figure S4: RFS after hepatectomy; Table S1: Pathological assessment of tumor regression grade (TRG), modified tumor regression grade (mTRG), dangerous halo (DH), and sinusoidal obstruction syndrome (SOS); Table S2: The operative procedure; Table S3: Postoperative complications; Table S4: Differences in tumor regression grade (TRG), modified tumor regression grade (mTRG), dangerous halo (DH), and sinusoidal obstruction syndrome (SOS); Table S5: Relationships between pathological response (TRG) and radiological response (RECIST); Table S6: Relationships between pathological response (mTRG) and radiological response (RECIST).
