# Supplementary material for: Pathological Evaluation of Resected Colorectal Liver Metastases: mFOLFOX6 Plus Bevacizumab versus mFOLFOX6 Plus Cetuximab in the Phase II ATOM Trial
Source: Cancers (Basel). 2022 Sep 9;14(18):4392. doi: 10.3390/cancers14184392 (PMC9496839; doi:10.3390/cancers14184392)
Supplement: Supplementary file 1 [file cancers-14-04392-s001.zip › cancers-1830176-supplementary.pdf]

Supplementary Materials

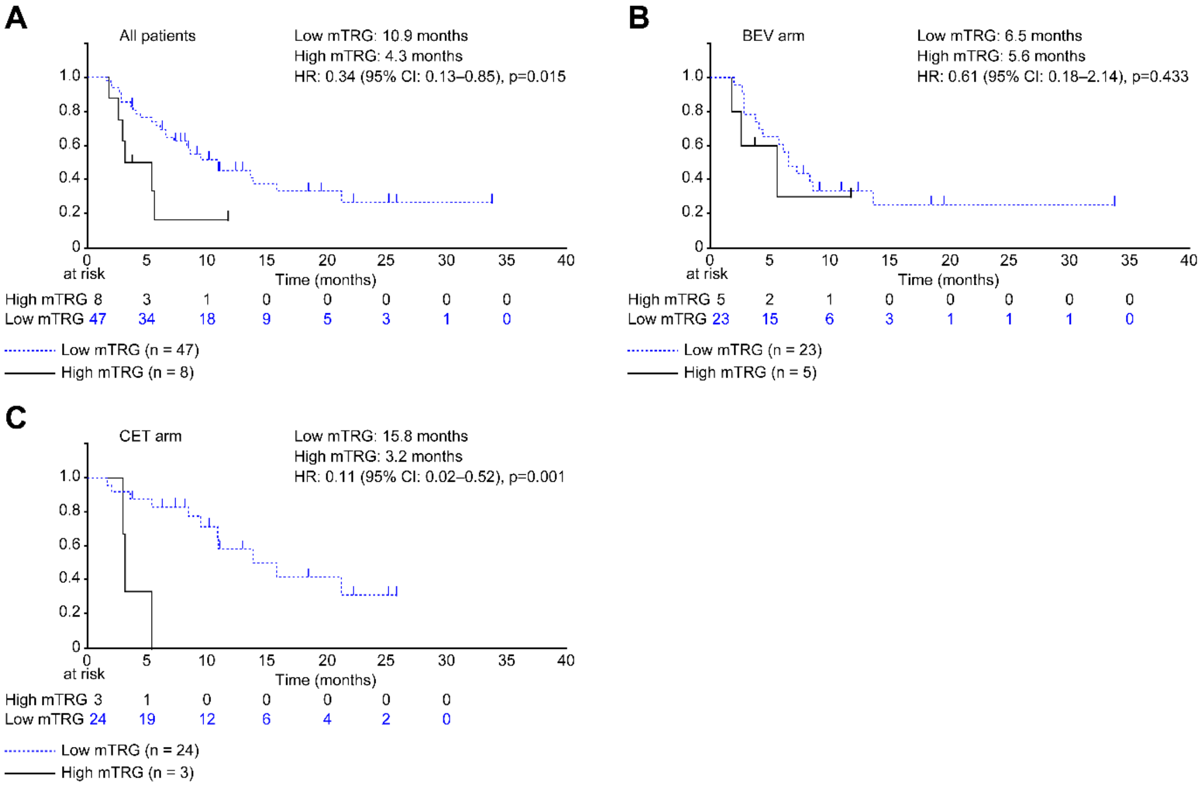

Supplementary Figure S1. Kaplan–Meier curves for relapse-free survival (RFS, time to relapse after resection) according to the modified tumor regression grade (mTRG). Curves are shown for all patients (A), patients in the bevacizumab (BEV) arm (B), and patients in the cetuximab (CET) arm (C). Relative to patients with high mTRG, patients with low mTRG had significantly better RFS (median: 4.3 months vs. 10.9 months; hazard ratio [HR]: 0.34, 95% confidence interval [CI]: 0.13–0.85; P = 0.015). Similarly, relative to high mTRG, low mTRG was associated with insignificantly better RFS in the BEV arm (median: 5.6 months vs. 6.5 months; HR: 0.61, 95% CI: 0.18–2.14; P = 0.433) and significantly better RFS in the CET arm (median: 3.2 months vs. 15.8 months; HR: 0.11, 95% CI: 0.02–0.52; P = 0.001).

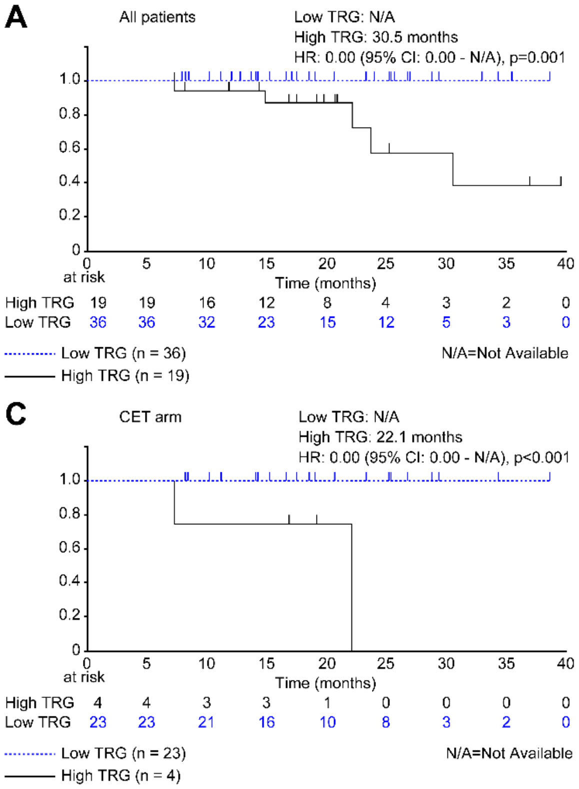

Supplementary Figure S2. Kaplan–Meier curves for overall survival (OS) based on the tumor regression grade (TRG) among all patients (A), patients in the bevacizumab (BEV) arm (B), and patients in the cetuximab (CET) arm (C).

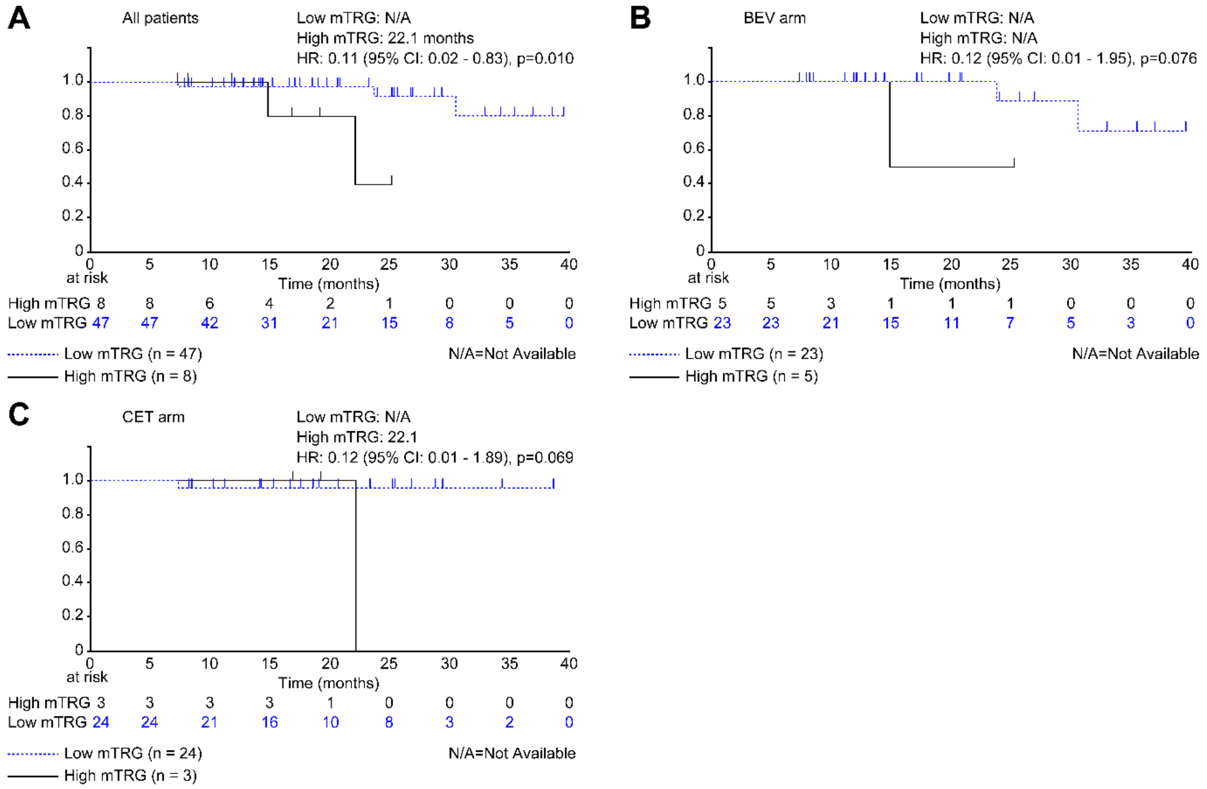

Supplementary Figure S3. Kaplan–Meier curves for overall survival (OS) based on the modified tumor regression grade (mTRG) among all patients (A), patients in the bevacizumab (BEV) arm (B), and patients in the cetuximab (CET) arm (C).

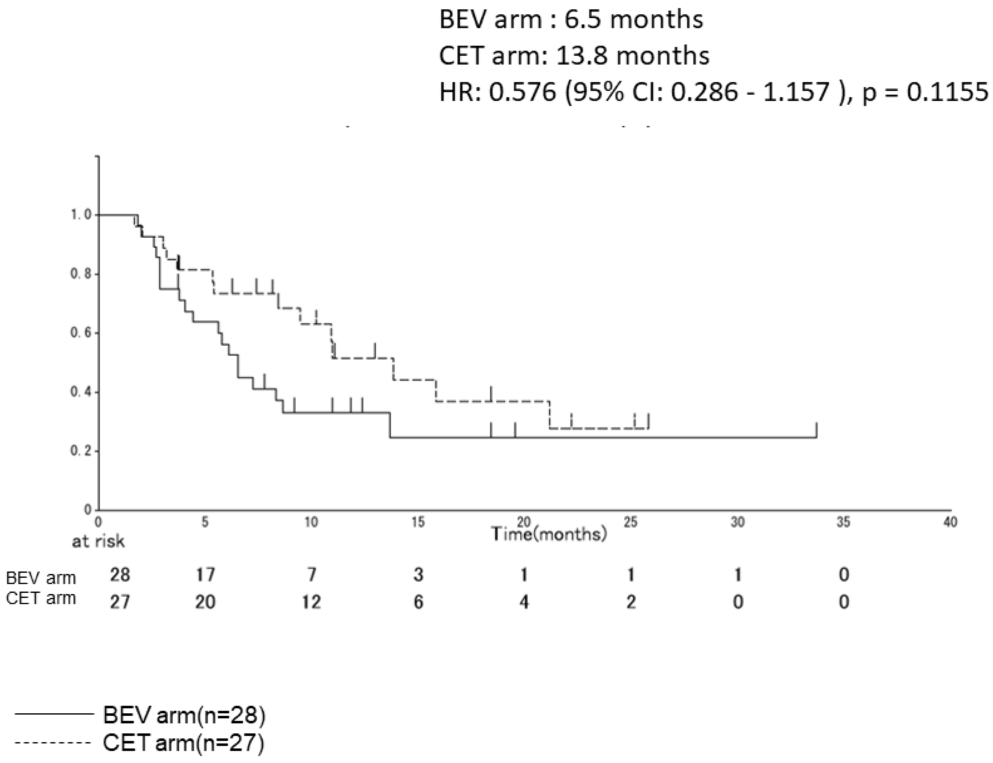

Supplementary Figure S4. RFS after hepatectomy

Supplementary Table S1. Pathological assessment of tumor regression grade (TRG), modified tumor regression grade (mTRG), dangerous halo (DH), and sinusoidal obstruction syndrome (SOS)

Tumor regression grade (TRG)	
TRG1	no viable tumor cells; area replaced by fibrosis
TRG2	mostly abundant fibrosis with a small number of viable tumor cells
TRG3	predominantly fibrosis with more viable tumor cells than in TRG2; tumor cells less than fibrosis
TRG4	more tumor cells than fibrosis
TRG5	only viable tumor cells

Modified tumor regression grade (mTRG)[*]	
mTRG1	no viable tumor cells; area replaced by fibrosis/ILN
mTRG2	mostly abundant fibrosis and ILN with a small number of viable tumor cells
mTRG3	mainly fibrosis and ILN, but a larger number of viable tumor cells when compared to mTRG2; tumor cells less than fibrosis and ILN
mTRG4	more tumor cells than fibrosis and ILN
mTRG5	only viable tumor cells

[*]mTRG considers the presence of infarct-like necrosis (ILN), which is caused by chemotherapy and presents as eosinophilic homogenous necrosis with no nuclear debris, which is surrounded by hyalinized fibrosis with histiocytic infiltration.

Dangerous halo (DH)[†]	
absent	no cluster of tumor cells
rare	scattered tumor cells infiltrating the liver parenchyma for < 10% of the lesion's circumference
focal	scattered cells infiltrating the liver parenchyma for 10–50% of the lesion's circumference
diffuse	scattered cells infiltrating the liver parenchyma for > 50% of the lesion's circumference

[†]DH is a cluster of tumor cells infiltrating the surrounding liver parenchyma without proliferating in the fibrotic stroma.

Sinusoidal obstruction syndrome (SOS)[‡]	
grade 1	none/mild and localized around the central vein
grade 2	moderate and extends from zone 1 to zone 2
grade 3	the lobule is completely and severely involved

‡SOS shows varying degrees of centrilobular sinusoidal dilatation, congestion, and hemorrhage with atrophy and loss of hepatocytes.

Supplementary Table S2. The operative procedure

	All	BEV	CET
Operative procedure*	N=55	N=28	N=27
partial hepatectomy	41	21	20
metasectomy	1	0	1
subsegmentectomy	7	5	2
segmentectomy	16	7	9
right lobectomy	6	3	3
left lobectomy	5	3	2
left hepatic trisegmentectomy	2	2	0
Others	4	1	3

*Duplicate

Supplementary Table S3. Postoperative complications

	All(N=55)		BEV(N=28)		CET(N=27)	
Postoperative complications*	Any	≥III	Any	≥III	Any	≥III
Postoperative bleeding	1 (1.8%)	0	1 (3.6%)	0	0	0
Bile leakage	12 (21.8%)	9 (16.4%)	5 (17.9%)	4 (14.3%)	7 (25.9%)	5 (18.5%)
Intraperitoneal abscess	5 (9.1%)	3 (5.5%)	2 (7.1%)	1 (3.6%)	3 (11.1%)	2 (7.4%)
Liver abscess	1 (1.8%)	0	1 (3.6%)	0	0	0
ileus	2 (3.6%)	0	2 (7.1%)	0	0	0
Others	3 (5.5%)	0	0	0	3 (11.1%)	0

*JCOG Postoperative Complication Criteria according to Clavien-Dindo Classification

Supplementary Table S4. Differences in tumor regression grade (TRG), modified tumor regression grade (mTRG), dangerous halo (DH), and sinusoidal obstruction syndrome (SOS)

	TRG		
	All (n=55)	BEV arm (n=28)	CET arm (n=27)
TRG1	1 (1.8%)	0 (0%)	1 (3.7%)
TRG2	15 (27.3%)	6 (21.4%)	9 (33.3%)
TRG3	20 (36.4%)	7 (25.0%)	13 (48.1%)
TRG4	15 (27.3%)	12 (42.9%)	3 (11.1%)
TRG5	4 (7.3%)	3 (10.7%)	1 (3.7%)

	mTRG		
	All (n=55)	BEV arm (n=28)	CET arm (n=27)
mTRG1	1 (1.8%)	0 (0%)	1 (3.7%)
mTRG2	21 (38.2%)	12 (42.9%)	9 (33.3%)
mTRG3	25 (45.5%)	11 (39.3%)	14 (51.9%)
mTRG4	7 (12.7%)	5 (17.9%)	2 (7.4%)
mTRG5	1 (1.8%)	0 (0%)	1(3.7%)

	DH		
	All (n=55)	BEV arm (n=28)	CET arm (n=27)
Absent	14 (25.5%)	4 (14.3%)	10 (37.0%)
Rare	19 (34.5%)	10 (35.7%)	9 (33.3%)
Focal	13 (23.6%)	6 (21.4%)	7 (25.9%)
Diffuse	9 (16.4%)	8 (28.6%)	1 (3.7%)

SOS			
	All (n=55)	BEV arm (n=28)	CET arm (n=27)
Grade 3	0	0	0
Grade 2	5 (9.1%)	0	5 (18.5%)
Grade 1	50 (90.9%)	28 (100%)	22 (81.5%)

TRG, tumor regression grade; mTRG, modified TRG; DH, dangerous halo; SOS, sinusoidal obstruction syndrome; BEV, bevacizumab; CET, cetuximab;

Supplementary Table S5. Relationships between pathological response (TRG) and radiological response (RECIST)

TRG	CR<br>n (%)	PR<br>n (%)	SD<br>n (%)	PD<br>n (%)	NE<br>n (%)	n (% [95% CI])	Fisher's exact test
All patients							
Low TRG	0 (0.0%)	36 (100.0%)	0 (0.0%)	0 (0.0%)	0 (0.0%)	36 (100.0% [90.3–100.0%])	<0.001
High TRG	0 (0.0%)	11 (57.9%)	8 (42.1%)	0 (0.0%)	0 (0.0%)	11 (57.9% [33.5–79.7%])	
BEV arm							
Low TRG	0 (0.0%)	13 (100.0%)	0 (0.0%)	0 (0.0%)	0 (0.0%)	13 (100.0% [75.3–100.0%])	0.0025
High TRG	0 (0.0%)	7 (46.7%)	8 (53.3%)	0 (0.0%)	0 (0.0%)	7 (46.7% [21.3–73.4%])	
CET arm							
Low TRG	0 (0.0%)	23 (100.0%)	0 (0.0%)	0 (0.0%)	0 (0.0%)	23 (100.0% [85.2–100.0%])	0.1412
High TRG	0 (0.0%)	4 (100.0%)	0 (0.0%)	0 (0.0%)	0 (0.0%)	4 (100.0% [39.8–100.0%])	

BEV, bevacizumab; CET, cetuximab; CI, confidence interval; CR, complete response; High TRG, TRG grades 4–5 (> 50% viable tumor cells); Low TRG, TRG grades 1–3 (≤ 50% viable tumor cells); NE, not evaluable; PD, progressive disease; PR, partial response; RECIST, Response Evaluation Criteria in Solid Tumors; SD, stable disease; TRG, tumor regression grade

Supplementary Table S6. Relationships between pathological response (mTRG) and radiological response (RECIST)

mTRG	CR n (%)	PR n (%)	SD n (%)	PD n (%)	NE n (%)	*N* (% [95% CI])	Fisher's exact test
All patients							
Low mTRG	0 (0.0%)	40 (85.1%)	7 (14.9%)	0 (0.0%)	0 (0.0%)	40 (85.1% [71.7–93.8%])	1.0000
High mTRG	0 (0.0%)	7 (87.5%)	1 (12.5%)	0 (0.0%)	0 (0.0%)	7 (87.5% [47.3–99.7%])	
BEV arm							
Low mTRG	0 (0.0%)	16 (69.6%)	7 (30.4%)	0 (0.0%)	0 (0.0%)	16 (69.6% [47.1–86.8%])	1.0000
High mTRG	0 (0.0%)	4 (80.0%)	1(20.0%)	0 (0.0%)	0 (0.0%)	4 (80.0% [28.4–99.5%])	
CET arm							
Low mTRG	0 (0.0%)	24 (100.0%)	0 (0.0%)	0 (0.0%)	0 (0.0%)	24 (100.0% [85.8–100.0%])	0.0084
High mTRG	0 (0.0%)	3 (100.0%)	0 (0.0%)	0 (0.0%)	0 (0.0%)	3 (100.0% [29.2–100.0%])	

BEV, bevacizumab; CET, cetuximab; CI, confidence interval; CR, complete response; High mTRG, mTRG grades 4–5 (> 50% viable tumor cells); Low mTRG, mTRG grades 1–3 (≤ 50% viable tumor cells); mTRG, modified tumor regression grade; NE, not evaluable; PD, progressive disease; PR, partial response; RECIST, Response Evaluation Criteria in Solid Tumors; SD, stable dise
